# Left Parietal Functional Connectivity Mediates the Association Between *COMT* rs4633 and Verbal Intelligence in Healthy Adults

**DOI:** 10.3389/fnins.2018.00233

**Published:** 2018-04-10

**Authors:** Qiang Xu, Jilian Fu, Feng Liu, Wen Qin, Bing Liu, Tianzi Jiang, Chunshui Yu

**Affiliations:** ^1^Tianjin Key Laboratory of Functional Imaging, Department of Radiology, Tianjin Medical University General Hospital, Tianjin, China; ^2^Brainnetome Center, Institute of Automation, Chinese Academy of Sciences, Beijing, China

**Keywords:** *COMT*, functional connectivity density, functional magnetic resonance imaging, single nucleotide polymorphism, verbal intelligence

## Abstract

In Chinese Han population, Catechol-O-methyltransferase gene (*COMT)* rs4633 is found to be associated with impaired cognitive process. We aimed to investigate the association between *COMT* rs4633 and verbal intelligence and the underlying neural mechanisms in Chinese Han healthy young adults. In 256 Chinese Han healthy young adults, we explored the modulatory effects of *COMT* rs4633 on verbal intelligence quotient (VIQ) and functional connectivity density (FCD) of the brain and the mediation effect of FCD on the association between *COMT* and VIQ. We further investigated the association between the expression patterns of dopamine receptor genes and the effect of *COMT* on FCD in the human brain. *COMT* rs4633 TT homozygotes exhibited lower VIQ than CC homozygotes and TC heterozygotes, higher long-range FCD (lrFCD) than CC homozygotes and TC heterozygotes in the left superior frontal gyrus. TT homozygotes and TC heterozygotes showed higher lrFCD than CC homozygotes in the left inferior parietal lobule. The lrFCD differences across genotypic subgroups were negatively associated with the expression of *DRD2* and *DRD3* genes. The left parietal lrFCD mediated the association between *COMT* rs4633 and VIQ. These findings provide a biological pathway that *COMT* rs4633 affects verbal intelligence via modulating the lrFCD of the left inferior parietal lobule.

## Introduction

The central dopaminergic system plays a critical role in human intelligence mainly via coordinating motor activity and influencing on cognitive skills (Guo et al., 2006). In the dopaminergic system, one of the most frequently studied enzymes, catechol-O-methyltransferase (COMT), which is encoded by *COMT* gene, can degrade synaptic dopamine (Männistö and Kaakkola, 1999). COMT activity is regulated by its genetic polymorphism and the *COMT* gene is one of the most promising candidate genes for human intelligence (Männistö and Kaakkola, 1999; Previc, 1999; Wang et al., 2009; Chen et al., 2016). *COMT* rs4633 risk T-allele is associated with susceptibility to impaired cognitive process in a Chinese Han population study. It may be induced by increased expression of the *COMT* gene, which leads to decreased dopamine level, lower functionality in certain brain regions, and bad cognitive performance (Wang et al., 2009).

Dopaminergic system plays an important role in abstract intellectual behaviors and language skills, both of which are important constitutions for human verbal intelligence (Guo et al., 2006; Schwartz and Beaver, 2013). One study shows that striatal D_2_/D_3_ receptor availability correlated positively with verbal intelligence quotient (VIQ) rather than performance intelligence quotient (PIQ) (Guo et al., 2006). These above pieces of evidence suggest that dopamine system may play a critical role in human verbal intelligence, however, the neural mechanism mediating effect of dopaminergic genes on verbal intelligence is unknown.

According to the parieto-frontal integration theory (P-FIT) of intelligence, many distributed brain areas rather than a single area involve in the intelligence formation, predominantly the left parietal and frontal lobes (Jung and Haier, 2007). What's more, neural efficiency hypothesis of intelligence also claims that individuals with higher intelligence have higher efficiency and lower energetic demands in brain network, implying that brain regions with energy-efficient hubs support higher cognitive performance (Jung and Haier, 2007; Li et al., 2009; Tomasi and Volkow, 2010). As a result, intelligence could be better characterized by imaging measures reflecting whole brain functional integration. Functional connectivity density (FCD) mapping is a graph theory-based tool for exploring topology of brain function using resting-state magnetic resonance imaging (MRI) data (Tomasi et al., 2016). It has several advantages over traditional graph theory methods. Firstly, FCD mapping could quantify local and global degree voxel-wisely with high resolution (3 mm isotropic) in an ultra-fast way (Tomasi and Volkow, 2010, 2011; Tomasi et al., 2016). Besides, compared with functional connectivity strength method, which reflects one voxel-to -one voxel relationship, FCD measures one voxel-to-many voxels relationships.(Zhuo et al., 2014) Secondly, the distribution of nodes in brain function corresponds with some traditional graph theory metrics, such as small world (Watts and Strogatz, 1998) and scale-free network (Barabasi and Albert, 1999). Thirdly, traditional graph theory-based method actually needs prior knowledge to parcellate whole brain into different brain regions by using user-defined brain atlas template, which may bias graph theory metrics using varying templates. Finally, FCD metrics have been extensively investigated in metabolic cost (Tomasi et al., 2014) and genetic influences of dopamine genes (Tian et al., 2013), suggesting it is a valid tool to depict the genetic influence on brain function. Based on the relative spatial distance relations between correlated voxels, the global FCD can be further divided into short- and long-range FCDs (Tomasi and Volkow, 2012). Greater FCD values for particular voxels reflect higher energy demands for functional connectivity and a more important role in the information processing (Tomasi et al., 2013).

Based on abovementioned findings, we could hypothesize that *COMT* rs4633 modulates verbal intelligence and functional connectivity in human brains, especially in left parietal and frontal lobes, which may mediate the effect of *COMT* rs4633 on verbal intelligence. In the present study, we aimed to test these hypotheses in healthy Chinese Han adults. Firstly, we compared verbal intelligence differences among the three *COMT* rs4633 genotypic groups (CC, TC, TT), afterwards we compare FCD differences among these three *COMT* rs4633 genotypic groups. Then, we build the gene-brain-cognition pathway. Furthermore, by using Allen Human Brain Atlas (AHBA), we investigated the relationship between expression patterns of two dopamine receptor genes (dopamine receptor 2 gene, *DRD2*; dopamine receptor 3 gene, *DRD3*) associated with verbal intelligence (Chen et al., 2005; Guo et al., 2006) and statistical map of group differences of FCD.

## Materials and methods

### Subjects

The experimental protocol was approved by the Medical Research Ethics Committee of Tianjin Medical University General Hospital, and written informed consent was obtained from all subjects. We recruited 323 right-handed healthy young adults (age: 22.7 ± 2.5 years; 157 males and 166 females). All participants were carefully screened to make sure that they had neither a history of psychiatric or neurological illness, nor drug or alcohol abuse. We only included Chinese Han subjects, and those who have contraindications for MRI examinations were excluded. Chinese edition of the Edinburgh Handedness Inventory (Oldfield, 1971) were assessed to ensure all participants were strong right-handed.

### Cognitive assessments

The full scale, verbal and performance intelligence quotients (IQ) were assessed using the Chinese Revised Wechsler Adult Intelligence Scale (WAIS-RC). The executive function was evaluated by the percentage of perseverative errors (PPE) derived from the Wisconsin Card Sorting Test. The working memory capacity was assessed using the 2 and 3-back accuracy. Details of these task procedures have been previously described (Gong, 1982; Heaton, 1993; Ding et al., 2012).

### Genotyping

The EZgene^TM^ Blood gDNA Miniprep Kit (Biomiga, Inc., San Diego, CA, USA) was used to extract genomic DNA from the whole blood. The genotypes of *COMT* rs4633 and rs4680 (Genotyping method for *COMT* rs4680 was described in our previous study; Tian et al., 2013) were obtained from each subject using the polymerase chain reaction (PCR) and ligation detection reaction (LDR) method (Thomas et al., 2004; Yi et al., 2009). We genotyped *COMT* rs4680 for calculating linkage disequilibrium (LD) between *COMT* rs4633 and rs4680 by using software Haploview v4.2 (Barrett et al., 2005) due to complete LD between these two single nucleotide polymorphisms (SNPs) in European population. The forward sequence of PCR primer sequences for *COMT* rs4633 was 5′-CTCTGCTGTTGGCAGCTGTGT-3′; and the reverse sequence was 5′-GTTCATGGCCCACTCCTTCTG-3′. 29 subjects of 323 were excluded from further analysis due to genotyping failure. We divided subjects into CC, TC, and TT subgroups according to their genotypes. Detailed method for genotyping of *COMT* rs4633 please see Supplementary Material.

### Imaging data acquisition

MRI data were obtained using a 3.0-Tesla MR system (Signa HDx 3.0 Tesla MR scanner, General Electric, Milwaukee, WI, USA). Resting-state functional images were acquired with a single-shot gradient echo planar imaging sequence, which is sensitive to blood oxygenation level-dependent (BOLD) contrast with the following parameters: repetition time (TR) = 2,000 ms; echo time (TE) = 30 ms; field of view (FOV) = 240 mm × 240 mm; matrix = 64 × 64; flip angle (FA) = 90°; slice thickness = 4 mm, no gap; 40 interleaved transversal slices and 180 volumes, lasting for 6 min. Sagittal 3D T1-weighted images were collected using a brain volume sequence with the following parameters: TR/TE = 8.1/3.1 ms; inversion time = 450 ms; FA = 13°; FOV = 256 mm × 256 mm; matrix = 256 × 256; slice thickness = 1 mm, no gap; and 176 sagittal slices. During the fMRI scans, all participants were instructed to move as little as possible, keep their eyes closed, think of nothing in particular and refrain from sleeping. After scanning, questionnaire was used to assess their waking state during fMRI scans.

### fMRI data preprocessing

Using the SPM12 (www.fil.ion.ucl.ac.uk/spm), the preprocessing of resting-state fMRI data included the following steps: deletion of first 10 volumes, slice timing, realignment for head motion, normalization, band-pass filtration, and regressing nuisance covariation. Detailed procedures please see Supplementary Material.

### FCD calculation

The FCD of each voxel was computed using an in-house script written in Linux platform according to the method described previously (Tomasi and Volkow, 2011). Functional connections were calculated using Pearson's linear correlation, and a functional connection existed if the correlation coefficient between any two voxels > 0.6. This threshold was recommended to be the optimum for calculating FCD (Tomasi and Volkow, 2012). The global FCD of a specific voxel x_0_ was defined as the total number of functional connections between x_0_ and all other voxels. The short-range FCD of a given voxel x_0_ was calculated as the total number of directly and indirectly neighboring voxels that were functionally connected with x_0._ The long-range FCD (lrFCD) was equaled to the global FCD minus the short-range FCD and it reflected the number of non-neighboring voxels of x_0_ that were functionally connected to x_0_. In this study, we also computed FCD using *r* = 0.5 and *r* = 0.7 to validate the results. For the detail procedures for minimizing unwished effects for susceptibility-related signal-loss artifacts, improvement of the normality of data distribution and spatial smoothness, please see Supplementary Material.

### Statistical analyses

Demographic and behavior data were analyzed using the Statistical Package for the Social Sciences (SPSS 20.0, Chicago, IL, USA). Specifically, a Chi-square test was used to compare group differences for categorical data. One-way ANCOVA controlling for educational years and working memory accuracy was used to compare group differences on continuous data. Bonferroni method was applied to correct for multiple comparisons (*P* < 0.05).

With SPM12 software, one-way ANCOVA was used to voxel-wisely investigate FCD differences among genotypic groups while controlling for educational years and working memory accuracy. Multiple comparisons were corrected using Monte Carlo simulation with a corrected threshold of *P* < 0.05 [AlphaSim program in DPABI software (Yan et al., 2016) with the following parameters: a voxel-level *P* < 0.001, 5,000 simulations, estimated smoothing kernel]. For each significant cluster, we extracted the mean FCD values for each subject in regions of interest (ROIs) by drawing a sphere (*r* = 6 mm) centered at the peak MNI coordinates of the cluster. And then, *post-hoc* analysis was used to test FCD differences between genotypic subgroups (CC subgroup vs. TC subgroup, CC subgroup vs. TT subgroup, TC subgroup vs. TT group). Bonferroni method was used to correct for multiple comparisons (*P* < 0.05). Since there was an unbalanced sample size between these genotypic subgroups, a validation of voxel-wise analysis was made using nonparametric permutation in DPABI software (Yan et al., 2016) (the same voxel *P* < 0.001, permutation 10,000 times).

To verify the statistical power of our study, we did power analysis for each ROI exhibiting significant FCD difference among these three genotypic groups using G^*^Power 3 software (Version 3.1.9.2, program written by University Kiel, Germany).

### Mediation analysis

In statistics, a mediation model is served to clarify the underling relationship between the independent and dependent variables via one mediator or more. The conventional mediation analysis was first proposed by Baron and Kenny (Baron and Kenny, 1986) using causal step regression theory. Specifically, mediation effect existed if (1) X (independent variable) significantly predicted Y (dependent variable) (c ≠ 0); (2) X significantly predicted M (mediator variable) (a ≠ 0); and (3) M significantly predicted Y controlling for X (b ≠ 0). These criteria were assessed by testing the following equations:

(1) Y=i1+cX 

(2) M=i2+aX

(3) Y=i3+c′X+bM

However, it had some flaws, firstly, they stated that existence of main effect is the fundamental premise of conventional mediation analysis, whereas it was not necessary to require the existence of main effect for mediation analysis showed by many following researches (Preacher and Hayes, 2004; Zhao et al., 2010). Secondly, the conventional method did not directly test the significance of mediation path a × b (Zhao et al., 2010). What's more, we could not perform the mediation analysis for more than two independent variable using conventional mediation analysis, which was not adapted for our data due to three independent variables (TT, TC, and CC genotypic groups). Therefore, we used the up-to-date mediation analysis method (Preacher and Hayes, 2004; Zhao et al., 2010) by using PROCESS (http://www.processmacro.org/index.html) plugged-in SPSS 20.0, the test for significance of each path (a, b, and c) was no longer necessary to determine mediation effect of X on Y. Instead, we only need to test the significance of mediation path a × b (Hayes and Rockwood, 2016).

In our study, we defined the *COMT* rs4633 genotype as the independent variable, the mean FCD value of each ROI as the mediators, and the FIQ, VIQ, and PIQ as the dependent variables, while controlling for educational years, 2 and 3-back accuracy for both mediators and dependent variables. Parameters included model number = 4, bootstrap sample = 5,000, bootstrap confidence interval (CI) method = bias corrected, confidence level for CI = 90%. Because our independent variable was multiple-categorical (CC, CT, and TT genotypic groups) rather than dichotomous, relative indirect effect was tested by using indicator coding method with CC group as reference, transforming multiple-categorical variables (CC, CT, and TT) to two subgroups (D1 and D2). (D1 indicates TC relative to CC, D2 indicates TT relative to CC). 90% CI and effect size for relative indirect effect were reported for our mediation analysis.

### Gene expression analysis

To examine the association between the effect of *COMT* rs4633 on brain FCD and expression patterns of dopamine receptors, we performed sample-wise spatial correlation analysis to identify the relationship between statistical map derived from group difference of FCD and expression patterns of two dopamine receptors that have been previously implicated in verbal intelligence (Guo et al., 2006; Lumme et al., 2010) by utilizing publicly available gene expression data from six donated brains provided by AHBA (Hawrylycz et al., 2012). In brief, we extracted the MNI coordinates of each sample, then averaged values of the statistical map within each spherical ROI (*r* = 6 mm) centered at MNI coordinate. The average statistical values in ROIs were then spatially correlated with *DRD2* and *DRD3* gene expression values of across samples respectively. At last, one sample *T* test of correlation coefficient was used to compare the consistency among six brains. Detailed methods for gene expression analysis please see Supplementary Methods.

## Results

### Demographic and genotyping information in the FCD analysis

After excluding 44 subjects with genotyping failure (*n* = 29) and without cognitive data (*n* = 15), 279 subjects retained. Further excluding 23 subjects with ill image quality or excessive head motion, 256 subjects were included in the FCD analysis (Table 1). The genotypic distribution of *COMT* rs4633 in these subjects was in Hardy-Weinberg equilibrium (*P* = 0.492). We found no complete LD (*D*′ = 0.8, *R*^2^ = 0.56) between *COMT* rs4633 and rs4680 in our Chinese Han population. Significant difference across groups was present in years of education (*P* = 0.007), but not in gender and age (*P* > 0.05). We found similar results in 279 subjects, detailed demographic information in 279 subjects please see Supplementary Material.

**Table 1:** Demographic and behavioral data of the 256 participants in FCD analysis.

	***COMT*** **rs4633 genotypic group**			***F/*χ^2^ value**	***P*-value**
	**CC (*n* = 116)**	**TC (*n* = 109)**	**TT (*n* = 31)**		
Age (years)	22.75(2.355)	22.65(2.522)	22.61(2.431)	0.064	0.938
Education (years)	15.89(1.883)	15.61(2.211)	14.58(1.893)	5.070	0.007
Gender (M: F)	46/70	51/58	17/14	2.674	0.263
FIQ	118.91(7.854)	116.83(8.712)	111.71(11.004)	8.618	2.39 × 10^−4^
VIQ	119.37(8.446)	117.89(9.492)	110.65(13.331)	10.171	5.6 × 10^−5^
PIQ	113.97(9.479)	112.02(10.680)	110.61(10.465)	1.811	0.166
2-back accuracy	89.53(5.38)	88.35(5.50)	86.95(5.46)	3.178	0.043
3-back accuracy	82.77(6.21)	81.49(6.52)	79.32(6.19)	3.852	0.023
PPE	16.52(9.69)	14.94(8.94)	18.68(11.85)	1.989	0.139
FD	0.08(0.04)	0.09(0.05)	0.09(0.03)	1.414	0.245

*Data are presented as mean (standard deviation), otherwise as indicated. COMT, catechol-O-methyltransferase; F, female; FD, framewise displacement; FIQ, full-scale intelligence quotient; M, male; PIQ, performance intelligence quotient; VIQ, verbal intelligence quotient; PPE, percentage of perseverative errors*.

### Cognitive analysis

**Figure 1:**
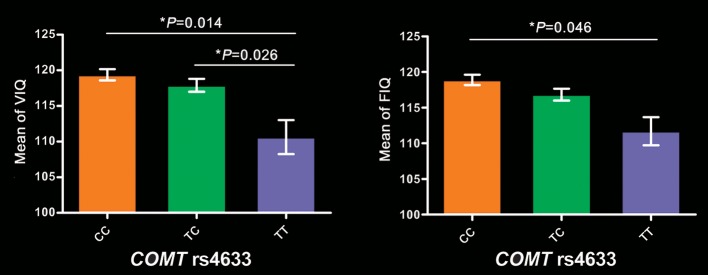
Intelligence differences among *COMT* genotypes in 256 participants. Bar plots depict the mean value and standard error. ^*^P < 0.05. *COMT*, catechol-O-methyltransferase; FIQ, full-scale intelligence quotient; VIQ, verbal intelligence quotient.

In these 256 subjects, *COMT* genotypic subgroups showed significant differences in VIQ (*P* = 5.6 × 10^−5^), FIQ (*P* = 2.39 × 10^−4^), 2-back (*P* = 0.043) and 3-back accuracy (*P* = 0.023), but not in PIQ (*P* = 0.166) and PPE (*P* = 0.139). After regressing out the effects of years of education, 2-back accuracy, and 3-back accuracy, VIQ (*P* = 0.014) and FIQ (*P* = 0.048) still significantly differed among these groups. Specifically, TT group exhibited lower VIQ than CC (*P* = 0.014) and TC (*P* = 0.026) groups, and lower FIQ than CC group (*P* = 0.046) (Figure 1). We also found significant frequency differences in genotype (*P* = 0.004) between high (VIQ ≥ 120) and normal (VIQ < 120) VIQ subgroups (χ^2^ = 11.081, *P* = 0.004). We found similar results in 279 subjects, detailed cognitive analysis results in 279 subjects please see Supplementary Results.

### Genotypic differences in short-range FCD

There was no significant difference (*P* > 0.05) in short-range FCD among the three *COMT* rs4633 genotypic groups.

### Genotypic differences in lrFCD

**Figure 2:**
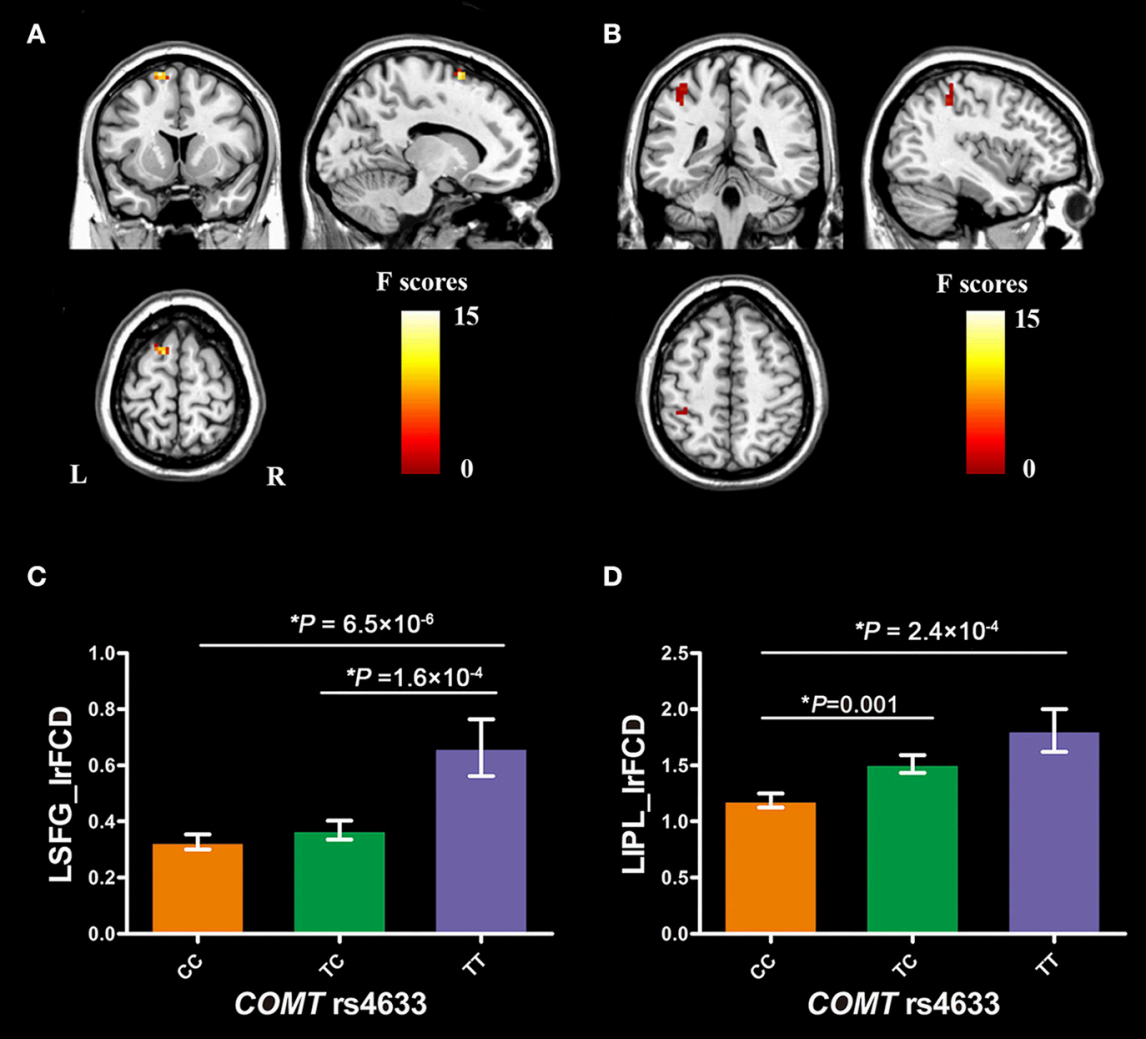
Brain regions with significant *COMT* genotypic difference on brain lrFCD and *COMT* genotypic differences in lrFCD of significant brain regions. **(A)** the left superior frontal gyrus; **(B)** the left inferior parietal lobule; **(C)** *COMT* genotypic differences in lrFCD of LSFG; **(D)** *COMT* genotypic differences in lrFCD of LIPL. Bar plots depict the mean value and standard error of lrFCD in each subgroup, ^*^P < 0.05. *COMT*, catechol-O-methyltransferase; L, left; R, right; LSFG, left superior frontal gyrus; LIPL, left inferior parietal lobule; lrFCD, long range functional connectivity density.

*COMT* rs4633 showed significant genotypic differences in the lrFCD in the left superior frontal gyrus (SFG, peak MNI coordinates: *x* = −15, *y* = 12, *z* = 69, cluster size = 19 voxels, peak *F* = 14.25, Figure 2A) and inferior parietal lobule (IPL, peak MNI coordinates: *x* = −39, *y* = −42, *z* = 48, cluster size = 20 voxels, peak *F* = 8.47, Figure 2B) after regressing out educational years, 2 and 3-back accuracy.

The mean FCD values of the two spherical ROIs (radius = 6 mm) were extracted from each subject. The *post-hoc* analysis showed that TT homozygotes had higher lrFCD than CC homozygotes (*P* = 6.5 × 10^−6^, Bonferroni corrected) and TC heterozygotes (*P* = 1.6 × 10^−4^, Bonferroni corrected) in the left SFG (Figure 2C). In the left IPL, TT homozygotes (*P* = 2.4 × 10^−4^, Bonferroni corrected) and TC heterozygotes (*P* = 0.001, Bonferroni corrected) had higher lrFCD than CC homozygotes (Figure 2D).

### Validation of FCD results

We firstly validated the voxel-wise results using nonparametric permutation test after controlling the same covariates, results were shown in Figure S2. Then, to validate our results derived from the connection threshold of 0.6, we also repeated the lrFCD analysis (AlphaSim corrected *P* < 0.05, voxel *P* < 0.001) using the connection thresholds 0.7 and 0.5 after controlling the same covariates. For the threshold of 0.7, the genotypic effects of *COMT* rs4633 on lrFCD were not significant: SFG (peak MNI coordinates: *x* = −15, *y* = 12, *z* = 51, cluster size = 8 voxels, peak *F* = 9.35) and IPL (peak MNI coordinates: *x* = −42, *y* = −42, *z* = 48, cluster size = 8 voxels, peak *F* = 8.09). For the threshold of 0.5, the genotypic effects of *COMT* rs4633 on lrFCD were also not significant: SFG (peak MNI coordinates: *x* = −12, *y* = 9, *z* = 66, cluster size = 11 voxels, peak *F* = 10.61) and IPL (peak MNI coordinates: *x* = −54, *y* = −51, *z* = 33, cluster size = 12 voxels, peak *F* = 9.29).

### Power analysis

For each cluster with significant genotypic effect, the statistical power was 0.9996 (medium effect size with *f* = 0.365) for the left SFG analysis and 0.9973 (medium effect size with *f* = 0.320) for the left IPL analysis (Please see Table S2).

### Mediation analysis

**Figure 3:**
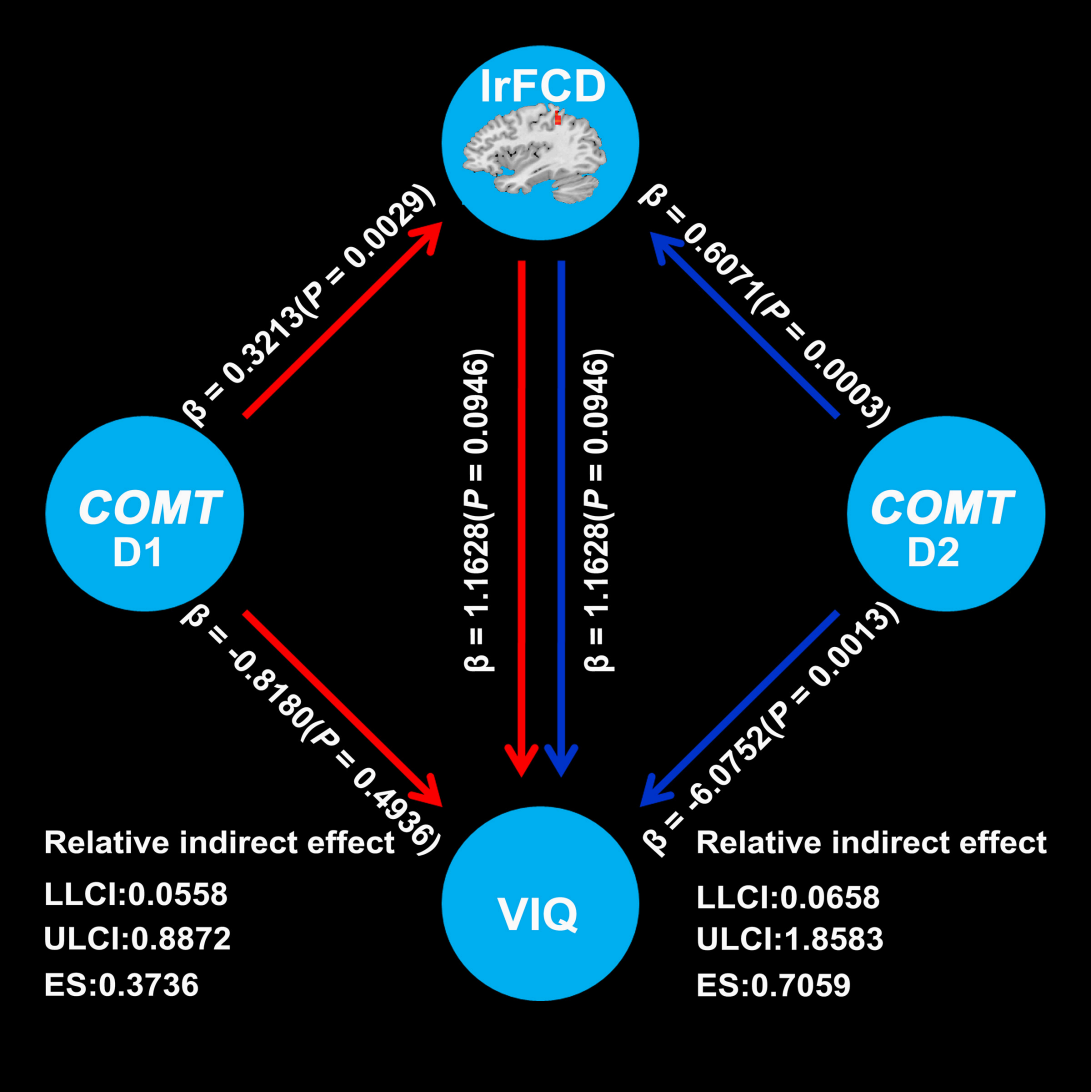
Mediation effects of lrFCD in the left inferior parietal lobule on the association between *COMT* rs4633 D1 and VIQ as well as D2 and VIQ. D1 means TC relative to CC, D2 means TT relative to CC; *COMT*, catechol-O-methyltransferase; VIQ, verbal intelligence quotient; LLCI, lower limit of confidence interval; ULCI, upper limit of confidence interval; ES, effect size. lrFCD, long range functional connectivity density.

Mediation analysis showed a significant indirect effect from COMT to VIQ mediated by the lrFCD of the left IPL (D1: 90% CI, 0.0558–0.8872; effect size = 0.3736; D2: 90% CI, 0.0658–1.8583; effect size = 0.7059) (Figure 3). These two confidential intervals did not contain zero, suggesting that mediation path a × b was significantly different from zero and consequently confirmed the existence of a mediation effect of the lrFCD of the left IPL on the association between *COMT* and VIQ. However, we did not find any mediation effect of the lrFCD of the left SFG.

### Association between FCD effect of *COMT* and dopamine-related gene expression

**Figure 4:**
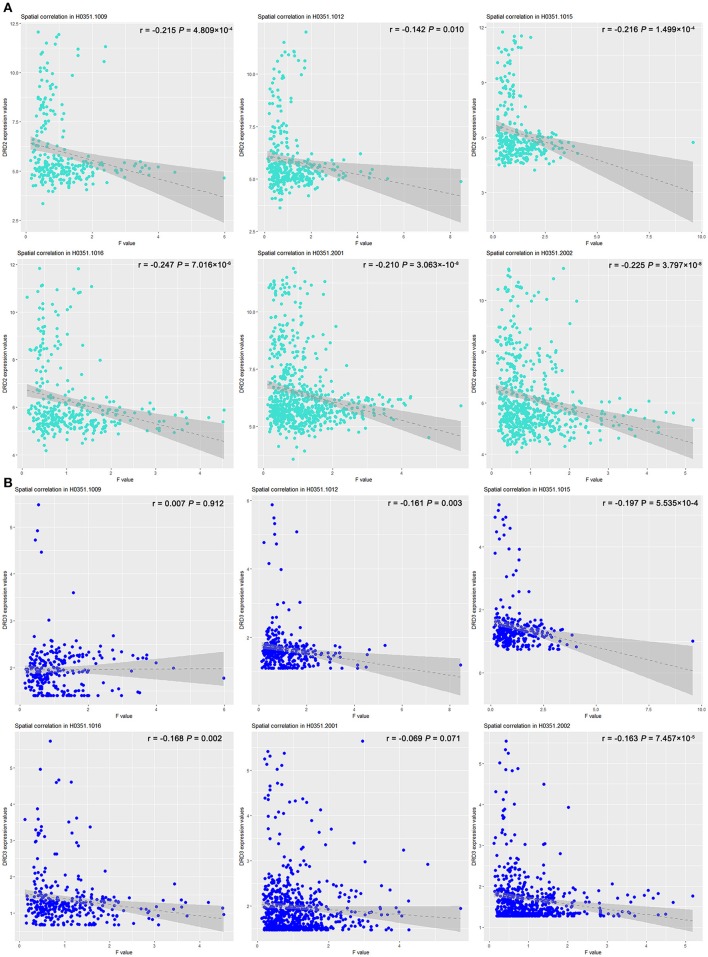
Scatter plots of spatial correlation between statistical map derived from group difference of lrFCD and two dopamine receptor genes. Scatter plots depict relations between expression values of **(A)** *DRD2* and **(B)** *DRD3* genes (y axis) and F-statistic value (x axis) corresponding to *COMT* rs4633 genotypic effect on lrFCD. *COMT*, catechol-O-methyltransferase; lrFCD, long range functional connectivity density.

The effect of *COMT* rs4633 on the lrFCD spatially correlated with the expression patterns of *DRD2* (*T* = –14.099*; P* = 6.460 × 10^−5^, Bonferroni corrected) and *DRD3* (T = –3.93; *P* = 0.022, Bonferroni corrected). The correlation coefficient and significance of each brain please see Figure 4 and Table S3.

## Discussion

In this study, we investigated gene-brain-cognition pathway to explore the neural mechanism mediating the genotypic effect of *COMT* rs4633 on verbal intelligence. We found significant genotypic effects of *COMT* rs4633 on verbal IQ and lrFCD in the left inferior parietal lobule and superior frontal gyrus. The mediation analysis revealed that the *COMT* rs4633 effect on verbal intelligence via modulating the lrFCD in left inferior parietal lobule. Finally, the statistical map of group difference of lrFCD was significantly associated with expression patterns of *DRD2* and *DRD3*.

### Genotypic effect of *COMT* rs4633 on verbal intelligence

Dopaminergic system plays an important role in human verbal intelligence formation (Previc, 1999; Guo et al., 2006; Lumme et al., 2010). Even though *COMT* rs4633 is a synonymous variation locating within exon 3 of the MB-COMT transcript and exon 1 of the S-COMT transcript, which do not result in alteration of amino acid sequence, its primary transcriptional mRNA might influence the *COMT* translation via changing RNA stem-loop structure (Nackley et al., 2006). Another possible mechanism for influencing expression of *COMT* might be due to cis-regulatory process which has an effect on transcription rate or transcript stability in an allele-specific way (Wittkopp et al., 2004). Specifically, whether in Chinese or Caucasian populations, there exist evidence that *COMT* rs4633 T allele is associated with increased *COMT* mRNA expression in peripheral blood cells or brain tissues, in comparison with CC genotype (Bray et al., 2003; Wang et al., 2009; Ramasamy et al., 2014). Furthermore, the increased expression of *COMT* in T-allele carriers may result in decreased dopamine level and hypofunction in certain brain regions, resulting in bad cognitive performance. Another evidence shows that left striatal D_2_/D_3_ receptor availability only correlates with VIQ, but not PIQ (Guo et al., 2006). These may explain our findings that TT homozygotes showed the lowest working memory accuracy and VIQ, not PIQ, in our population.

### The left parietal connectivity mediates the effect of *COMT* rs4633 on verbal intelligence

We find that TT carriers have a significant greater lrFCD than CC carriers in the left superior frontal gyrus and inferior parietal lobule. What's more, only the lrFCD in the left inferior parietal lobule mediates the effect of COMT rs4633 on verbal intelligence. However, the finding of genetic effect on parietal lobe lrFCD is not significant by changing connection thresholds at the same correction threshold (Alphasim corrected *P* < 0.05 voxel *P* = 0.001, cluster size = 13). The possible reason is that at a lower threshold (*r* = 0.5) or higher threshold (*r* = 0.7), the total number of connected voxels disproportionately increases or decreases, however, the genetic effect on lrFCD at threshold *r* = 0.5 and 0.7 highly spatially correlates (*r* = 0.77/0.85) with that of threshold *r* = 0.6. Besides, the number of survived voxels at threshold *r* = 0.5 (number of voxel is 12) or 0.7 (number of voxel is 8) is only slightly lower than cluster size of Alphasim correction threshold. The FCD measures the number of functional connections per voxel and is an ideal imaging measure of functional integration in the human brain. As illustrated by the parieto-frontal integration theory (P-FIT), the human intelligence depends on the distributed brain areas and their connections, among which the left prefrontal and parietal areas play a critical role in intelligence (Jung and Haier, 2007). Previous studies suggest that parietal lobe plays an important role in verbal intelligence (Weinstein and Teuber, 1957; Koenigs et al., 2009; Brownsett and Wise, 2010), which may explain why only the parietal lobule mediated the genotypic effect of COMT rs4633 on verbal intelligence. It seems odd that the COMT rs4633 TT subgroup had a lower dopamine level and showed a lower verbal intelligence, whereas had a higher lrFCD than CC subgroup. This may be explained by the intelligence efficiency hypothesis that individuals with higher intelligence needs lower energetic demands and vice versa (Jung and Haier, 2007; Li et al., 2009). Because a higher FCD has been associated with a greater glucose metabolism and corresponds to a larger energetic demands (Tomasi et al., 2013), which may account for why *COMT* rs4633 TT subjects with a lower verbal intelligence but showed a higher FCD.

### Possible biological mechanisms underlying *COMT* effect

Although we establish a system-level pathway from *COMT* rs4633 to the FCD in the left inferior parietal lobule then to verbal intelligence in Chinese Han healthy young adults, the exact modulation of gene on brain function remains unclear. Previous studies show that the dopamine acts on dopamine D_2_ and D_3_ receptors to regulate cognitive functions (Chen et al., 2005; Guo et al., 2006; Lumme et al., 2010). In this study, we find that brain regions with greater lrFCD differences among the three *COMT* genotypic groups show relatively lower expression of the *DRD2* and *DRD3*. D_2_ and D_3_ receptors belong to D_2_-like families, which are inhibitory receptors (Missale et al., 1998). That is to say, the lrFCD differences between *COMT* genotypes negatively correlated with the expression of the inhibitory dopamine receptors. It has been shown that the striatal dopamine D_2_/D_3_ receptor availability positively correlated with verbal cognitive functions (Chen et al., 2005; Guo et al., 2006), which is consistent with our finding that the both of *DRD2* and *DRD3* gene expression were negatively associated with statistical map of group difference of lrFCD. Taking together, our findings may suggest that COMT rs4633 modulates lrFCD and further affects verbal intelligence in Chinese Han subjects. This system-level pathway may be associated with function of D2 and D3 receptors.

There are several limitations in our study. Firstly, although we tried to interpret the molecular mechanism underlying this gene-brain-cognition pathway, the gene expression data are from a different dataset, this conclusion should be further investigated. Secondly, we found the genotypic effect on lrFCD, however, this finding slightly varied by different connection threshold. Thirdly, we only included one SNP, which could not find other genetic effect, such as gene-gene interaction effect.

## Conclusion

In summary, using mediation analysis and gene expression analysis, we established the pathway from the *COMT* rs4633 to the llrFCD of the left inferior parietal lobule and to the verbal intelligence, and proposed that this pathway may depend on the expression of the D_2_ and D_3_ receptors. These findings may depict a comprehensive picture of *COMT* rs4633 effect and provide further knowledge for pathophysiological mechanism of schizophrenia.

## Author contributions

CY and TJ: designed the experiment; QX, JF, and BL: performed the experiments; QX, JF, WQ, and FL: analyzed the data; QX, JF, and FL: drafted the manuscript; WQ, CY, BL, TJ: revised the manuscript; All authors discussed the results and gave final approval of the version to be published.

### Conflict of interest statement

The authors declare that the research was conducted in the absence of any commercial or financial relationships that could be construed as a potential conflict of interest. The reviewer DRS and handling Editor declared their shared affiliation.
