# Supplementary material for: Left Parietal Functional Connectivity Mediates the Association Between COMT rs4633 and Verbal Intelligence in Healthy Adults
Source: Front Neurosci. 2018 Apr 10;12:233. doi: 10.3389/fnins.2018.00233 (PMC5902573; doi:10.3389/fnins.2018.00233)
Supplement: Supplementary file 1 [file Presentation1.pdf]

Supplemental Materials

Supplementary method

COMT rs4633 genotyping

The PCR was operated at a 20 μL volume using 1 μL genomic DNA, 0.4 μL primer mixture, 2 μL dNTPs, 0.6 μL Mg^{2+}, 2 μL buffer, 4 μL Q-Solution, and 0.3 μL Taq DNA polymerase. The amplification protocol included an initial denaturation and enzyme activation phase at 95°C for 15 mins, followed by 35 cycles of denaturation at 94°C for 30 secs, annealing at 59°C for 1 min and 30 secs, extension at 72°C for 1 min, and then a final extension at 72°C for 7 mins. Afterwards, PCR products were affirmed in 3% agarose gels stained with ethidium bromide to make sure the amount of DNA added to the LDR.

Three probes were developed for the LDR reactions, including a common probe (rs4633_modify: P-TGGTTCAGGATGCGCTGCTCCTTGGTTTTTTTTTTTTTTTTTTT-FAM) and two discriminating probes (rs4633_C: TTTTTTTTTTTTTTTTTTTTTCCCGGGCTCCGCATGCTGCAGC, rs4633_T: TTTTTTTTTTTTTTTTTTTTTTTCCCGGGCTCCGCATGCTGCAGCACA ACG) for two alleles of *COMT* rs4633. These reactions were carried out in a 10 μL mixture containing 1 μL buffer, 1 μL probe mix, 0.05 μL Taq DNA ligase, 1 μL PCR product, and 6.95 μL deionized water. The reaction program comprised an initial heating at 95 °C for 2 min followed by 35 cycles of 30 s at 94 °C and 2 min at 50 °C. We ceased the reactions by chilling the tubes in an ethanol-dry ice bath and adding 0.5 mL of 0.5 mM EDTA. Aliquots of 1 μL of the reaction products were blended with 1 μL of loading buffer (83% formamide, 8.3 mM EDTA and 0.17% blue dextran) and 1 μL ABI GS-500 Rox-Fluorescent molecular weight marker, denatured at 95 °C for 2 mins, and chilled rapidly on ice prior to being loaded on a 5 M urea-5% polyacrylamide gel and electrophoresed on an ABI 3100 DNA sequencer at 3000 V. Finally, we used the ABI Gene Mapper software to assay and quantify fluorescent ligation products.

fMRI data preprocessing

The resting-state fMRI data were preprocessed using the SPM12 (www.fil.ion.ucl.ac.uk/spm) with the following steps. The first 10 volumes were discarded for signal reaching equilibrium and participants adapting to the scanning noise. Acquisition time delays were corrected between slices for remaining 170 volumes and these volumes were realigned to the first volume to correct head motions. During the correction of head motion, we only included subjects with translational or rotational motion parameters lower than 2 mm or 2°. What’s more, we calculated the frame-wise displacement (FD), an index representing volume-to-volume changes in head position (Power et al., 2012). We obtained FD from the derivatives of the rigid

body realignment estimates that were used to realign functional MRI data (Power et al., 2012; 2013). These functional images were spatially normalized to Montreal Neurological Institute (MNI) space using steps described below: we first linearly co-registered individual structural images into the mean motion-corrected functional image; then we segmented the co-registered structural images into gray matter, white matter and CSF, meanwhile nonlinearly co-registered gray matter to the MNI space; and finally we normalized the motion-corrected functional volumes into the MNI space by using the parameters estimated during nonlinear co-registration. Then, we resampled the normalized functional volumes into a voxel size of $3 \times 3 \times 3$ mm^3. At last, functional images were band-pass filtered with a frequency range of 0.01-0.1 Hz and nuisance covariates (including six head motion parameters and average BOLD signals of the ventricular, and white matter) were regressed out.

FCD calculation

To minimize unwanted effects from susceptibility-related signal-loss artifacts, a gray matter (GM) mask was applied to restrict the calculation of the FCD to voxels only in the GM regions with a signal-to-noise >50 (Tomasi and Volkow, 2010). To enhance the normality of the distribution, grand mean scaling of FCD was obtained by dividing by the mean FCD value of the qualified voxels in the whole brain. Finally, the normalized FCD maps were spatially smoothed with a $6 \times 6 \times 6$ mm^3 full-width at half maximum (FWHM) Gaussian kernel.

Gene expression analysis

There were four brains only having left-hemispheres and two brains having two hemispheres. Each donated brain had MRI scans before being dissected, so each sample had MNI coordinate. And importantly, mRNA microarray analysis was done for each sample to depict the transcriptional profiles in human brain (detailed information please see http://help.brain-map.org/display/humanbrain/Documentation).

The steps actually performed in this analysis are as follows. Firstly, To get the *DRD2* and *DRD3* expression values in each sample of six donated brains, we chose one probe to represent gene expression of *DRD2* and *DRD3* by calculating which probe was most correlated with other probes as done in previous studies (Hawrylycz et al., 2015; Forest et al., 2017), which could make the results more reliable. Secondly, we extracted the average F values indicating group differences of FCD within spherical ROIs (radius = 6mm) centered at MNI coordinate of each sample in each brain. Thirdly, we performed sample-wise spatial correlation between gene expression profiles and effect of *COMT* on brain FCD for *DRD2* and *DRD3*. Lastly, we did one sample T test for correlation coefficient to compare the consistency among six donated brains. Because we performed two spatial correlation analyses, Bonferroni method was applied to correct for multiple comparisons ($P < 0.05$).

References:

Forest, M., Iturria-Medina, Y., Goldman, J.S., Kleinman, C.L., Lovato, A., Oros Klein, K., et al. (2017). Gene networks show associations with seed region connectivity. *Hum Brain Mapp* 38(6)**,** 3126-3140. doi: 10.1002/hbm.23579.

Hawrylycz, M., Miller, J.A., Menon, V., Feng, D., Dolbeare, T., Guillozet-Bongaarts, A.L., et al. (2015). Canonical genetic signatures of the adult human brain. *Nat Neurosci* 18(12)**,** 1832-1844. doi: 10.1038/nn.4171.

Power, J.D., Barnes, K.A., Snyder, A.Z., Schlaggar, B.L., and Petersen, S.E. (2012). Spurious but systematic correlations in functional connectivity MRI networks arise from subject motion. *Neuroimage* 59(3)**,** 2142-2154. doi: 10.1016/j.neuroimage.2011.10.018.

Power, J.D., Barnes, K.A., Snyder, A.Z., Schlaggar, B.L., and Petersen, S.E. (2013). Steps toward optimizing motion artifact removal in functional connectivity MRI; a reply to Carp. *Neuroimage* 76**,** 439-441. doi: 10.1016/j.neuroimage.2012.03.017.

Tomasi, D., and Volkow, N.D. (2010). Functional connectivity density mapping. *Proc Natl Acad Sci U S A* 107(21)**,** 9885-9890. doi: 10.1073/pnas.1001414107.

Supplementary results

Demographic information in 279 subjects

After excluding 44 subjects with genotyping failure (n=29) and without cognitive data (n=15), 279 healthy young Chinese Han subjects were included in the cognitive analysis. The genotypic distributions of *COMT* rs4633 was in Hardy-Weinberg equilibrium (P = 0.431). There was significant difference in years of education (P = 0.009), but not in gender and age (P > 0.05) among the three genotypic subgroups (Table S1).

Cognitive analysis in 279 subjects

COMT genotypic subgroups showed significant differences in VIQ ($P = 9\times10^{-6}$), FIQ ($P = 1.06\times10^{-4}$) and 3-back accuracy (P = 0.01), but not in PIQ (P = 0.195), 2-back accuracy (P = 0.094) and PPE (P = 0.226). After regressing out the effects of years of education and 3-back accuracy, VIQ (P = 0.004) and FIQ (P = 0.02) still significantly differed among these groups. The *post-hoc* analysis revealed that TT group had significantly lower VIQ than CC (P = 0.003) and TC (P = 0.035) groups, and lower FIQ than CC group (P = 0.026) (Figure S1). Using chi-square test, we found significant frequency differences in genotype (P = 0.001) between high (VIQ ≥ 120) and normal (VIQ < 120) VIQ subgroups (χ^2 = 14.175, P = 0.001).

Supplementary table

Table S1. Demographic and behavioral data of the 279 participants in cognitive analysis

	***COMT* rs4633 genotypic group**			**F/χ^2 value**	***P* value**
	**CC(n=123)**	**TC(n=120)**	**TT(n=36)**		
**Age(years)**	22.72(2.345)	22.65(2.595)	22.68(2.840)	0.025	0.975
**Education(years)**	15.85(1.869)	15.65(2.226)	14.64(2.127)	4.819	0.009
**Gender (M: F)**	50/73	59/61	22/14	2.567	0.079
**FIQ**	118.77(7.697)	116.32(9.194)	111.69(10.386)	9.461	1.06×10^{-4}
**VIQ**	119.24(8.358)	117.26(9.780)	110.31(12.439)	12.127	9×10^{-6}
**PIQ**	113.86(9.244)	111.78(11.027)	111.25(10.286)	1.647	0.195
**2-back accuracy**	89.34(5.74)	88.40(5.49)	87.13(5.50)	2.381	0.094
**3-back accuracy**	82.67(6.22)	81.81(6.41)	79.01(6.10)	4.699	0.01
**PPE**	16.32(9.55)	15.21(8.72)	18.27(11.64)	1.496	0.226

Note: data are presented as mean (standard deviation), otherwise as indicated. *COMT*, catechol-O-methyltransferase; F, female; FIQ,full-scale intelligence quotient; M, male; PIQ, performance intelligence quotient; VIQ, verbal intelligence quotient; PPE, percentage of perseverative errors.

Table S2. Power analysis of lrFCD differences among three genotypic groups in left superior frontal gyrus and left inferior parietal gyrus respectively.

**Left superior frontal gyrus**		**Left inferior parietal gyrus**	
F tests - ANOVA: Fixed effects, omnibus, one-way		F tests - ANOVA: Fixed effects, omnibus, one-way	
Analysis: Post hoc: Compute achieved power		Analysis: Post hoc: Compute achieved power	
Input: Effect size f	0.3649598	Input: Effect size f	0.3200973
α err prob	0.05	α err prob	0.05
Total sample size	256	Total sample size	256
Number of groups	3	Number of groups	3
Output:Noncentrality parameter λ	34.0980878	Output:Noncentrality parameter λ	26.2303441
Critical F	3.0314859	Critical F	3.0314859
Numerator df	2	Numerator df	2
Denominator df	253	Denominator df	253
Power (1-β err prob)	0.9997560	Power (1-β err prob)	0.9973155

Table S3. Correlation coefficient and *P* value for each donor of *DRD2* and *DRD3*

	DRD2		***DRD3***	
Donor ID	r/T	*P* value	r/T	*P* value
H0351.1009	-0.215	4.809×10^{-4}	0.007	0.912
H0351.1012	-0.142	0.010	-0.161	0.003
H0351.1015*	-0.216	1.499×10^{-4}	-0.197	5.535×10^{-4}
H0351.1016	-0.247	7.016×10^{-6}	-0.168	0.002
H0351.2001	-0.210	3.063×-10^{-8}	-0.069	0.071
H0351.2002	-0.225	3.797×10^{-8}	-0.163	7.457×10^{-5}
One sample T test	-14.099	6.460×10^{-5}, Bonferroni corrected	-3.93	0.022, Bonferroni corrected

Note: Donors H0351.1009, H0351.1012, H0351.1015 and H0351.1016 only have left hemispheres, donors H0351.2001 and H0351.2002 have two hemispheres. Asterisk indicates female brain.

Supplementary figures legends:

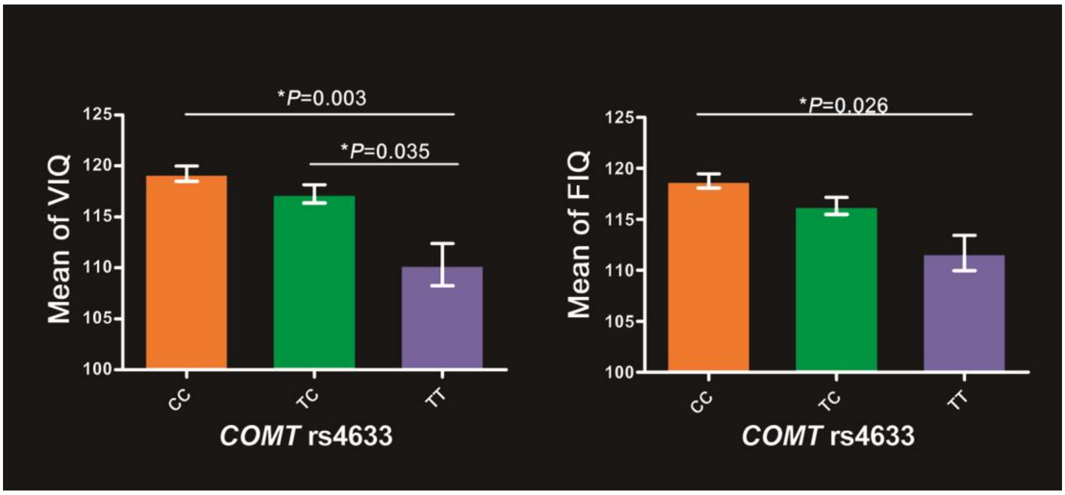

Figure S1. Intelligence differences among *COMT* genotypes in 279 subjects. Bar plots depict the mean value and standard error. **P* < 0.05.FIQ, *COMT*, catechol-O-methyltransferase; full-scale intelligence quotient; VIQ, verbal intelligence quotient.

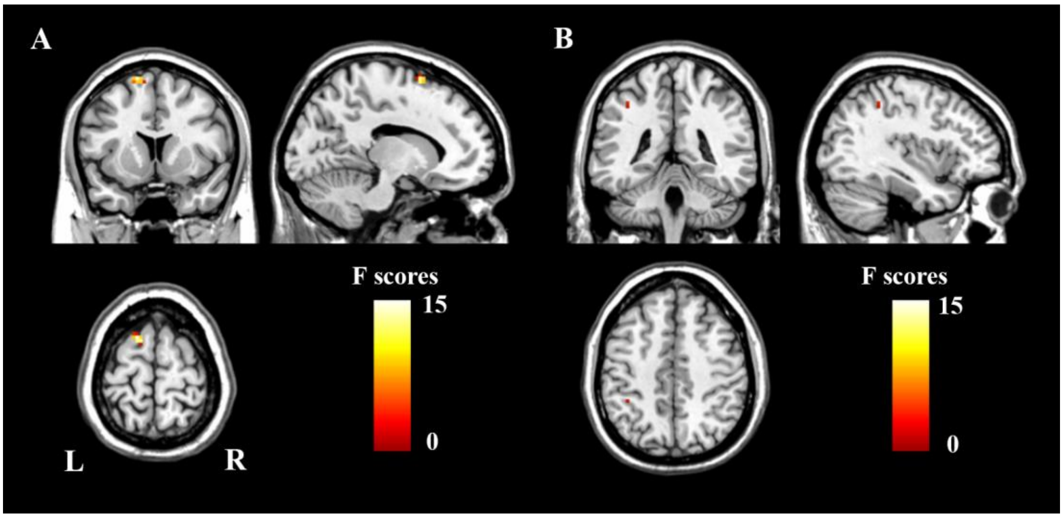

Figure S2. Brain regions with significant *COMT* genotypic difference on brain lrFCD with permutation test. (A) LSFG (peak MNI coordinates: x = −15, y = 12, z = 69, cluster size = 16 voxels, peak F =14.25); (B) LIPL (peak MNI coordinates: x = −39, y = -42, z = 48, cluster size = 2 voxels, peak F = 8.47). (Permutation test, 10,000 times, voxel *P* < 0.001). *COMT*, catechol-O-methyltransferase; L, left; R, right. LSFG, left superior frontal gyrus; LIPL, left inferior parietal lobule; lrFCD, long range functional connectivity density.
